# Bioelectrical impedance analysis to estimate one-repetition maximum measurement of muscle strength for leg press in healthy young adults

**DOI:** 10.1038/s41598-022-20526-8

**Published:** 2022-10-13

**Authors:** Keita Sue, Yukino Kobayashi, Mitsuru Ito, Maiko Midorikawa-Kijima, Shunichi Karasawa, Satoshi Katai, Kimito Momose

**Affiliations:** 1 Department of Rehabilitation, JA Nagano Kouseiren Kakeyu-Misayama Rehabilitation Center Kakeyu Hospital, 1308, Kakeyuonsen, Ueda, Nagano 386-0396 Japan; 2 Department of Health Sciences, Graduate School of Medicine, Science and Technology, Shinshu University, 3-1-1, Asahi, Matsumoto, Nagano 390-8621 Japan; 3 Department of Rehabilitation, Komaki Youtei Memorial Hospital, 1963, Chouda, Nisinoshima, Komaki, Aichi 485-0077 Japan; 4 Department of Radiology, JA Nagano Kouseiren Kakeyu-Misayama Rehabilitation Center Kakeyu Hospital, 1308, Kakeyuonsen, Ueda, Nagano 386-0396 Japan; 5 Department of Rehabilitation, Tums Urayasu hospital, 7-2-32, Takasu, Urayasu, Chiba, 279-0023 Japan; 6 Department of Physical Therapy, School of Health Science, Shinshu University, 3-1-1, Asahi, Matsumoto, Nagano 390-8621 Japan

**Keywords:** Skeletal muscle, Biomedical engineering, Health care

## Abstract

Resistance training (RT) progress is determined by an individual’s muscle strength, measured by one-repetition maximum (1RM). However, this evaluation is time-consuming and has some safety concerns. Bioelectrical impedance analysis (BIA) is a valid and easy-to-use method to assess skeletal muscle mass (SMM). Although BIA measurements are often correlated with muscle strength, few studies of 1RM for RT and BIA measurements are available. This observational study examined the relationship between 1RM and BIA measurements and developed BIA-based prediction models for 1RM. Thirty-five healthy young Japanese adults were included. SMM and the skeletal muscle mass index (SMI) were measured using the BIA device. In addition, dominant-leg 1RM was measured using a unilateral leg-press (LP) machine. The correlations between BIA measurements and 1RM were calculated, and simple regression analyses were performed to predict 1RM from the BIA variables. The results showed significant correlations between 1RM and dominant-leg SMM (R = 0.845, *P* = 0.0001) and SMI (R = 0.910, *P* = 0.0001). The prediction models for 1RM for LP derived from SMM of the dominant leg and SMI were Y = 8.21x + 8.77 (*P* = 0.0001), R^2^ = 0.73, and Y = 15.53x − 36.33 (*P* = 0.0001), R^2^ = 0.83, respectively. Our results indicated that BIA-based SMI might be used to predict 1RM for LP accurately.

## Introduction

Resistance training (RT) improves physical function and reduces the incidence of cardiovascular disease and all-cause mortality in adults^1^. In addition, multiple types of exercise, including RT, reportedly reduced fall risk in older adults^2^. Therefore, RT is an important physical activity to maintain health^3^. Particularly, RT progress is determined by one-repetition maximum (1RM), the maximum load a participant can lift at one repetition^4^. This measurement is useful for selecting strategies by either adopting heavier loads with fewer repetitions and sets^5^ or adopting moderate loads with additional repetitions and sets^6^.

Despite its time-consuming nature, direct 1RM measurement is a reliable strength evaluation method^7^. However, safety considerations are necessary because joint injuries in older adults^8^ and blood pressure elevations in young adults^9^ have been reported with the use of loads close to 1RM during RT. Therefore, for RT prescription, the development of safe 1RM measurement is important for individuals of all age groups. In addition, estimations from several repetitions performed with submaximal loads^10–12^ or maximal isometric muscle strength^13,14^ were reported as indirect 1RM measurements. Although these methods could shorten the time required to measure maximal loads, there are some concerns about cardiovascular safety^15^ because of the nearly maximal effort required for the estimation. Therefore, it is necessary to develop other safer methods for 1RM prediction.

Muscle strength is determined by a combination of morphological and neural factors^16^. Morphological factors such as skeletal muscle mass (SMM) and cross-sectional skeletal muscle area in the extremities have been well documented to have a moderate-to-strong correlation with muscle strength or power^17–19^, and evaluating these morphological factors might represent a viable option for muscle function assessment. Several methods can be used to accurately assess SMM, including magnetic resonance imaging, computed tomography, and dual-X-ray absorptiometry^20^. However, these modalities are not easily utilized because of their cost and environmental restrictions. Further, radiation exposure in computed tomography and dual-X-ray absorptiometry limits their use. In recent years, bioelectrical impedance analysis (BIA) has been widely used in both clinical and research settings. BIA is an easy-to-use, non-invasive, and relatively inexpensive method to assess body composition, including the SMM. The method uses alternating electrical current to measure the body’s resistance at a designed frequency and can estimate the body composition based on the differences in tissue-specific electrical conductivity. The validity of BIA for SMM measurement has been confirmed, particularly among Asian populations in previous studies^21–23^. In this regard, the Asian Working Group for Sarcopenia recommends its use for sarcopenia diagnosis, a condition characterized by progressive loss of muscle mass and strength upon aging^24^.

Some studies have reported moderate-to-strong correlations between muscle strength and BIA parameters^25–27^. However, most of the strength measurements used in previous studies involved isometric conditions^21,25–27^ instead of dynamic muscle strength (e.g., 1RM, which is essential for RT prescription). If the relationship between 1RM and parameters, such as SMM or skeletal mass index (SMI, an index of upper and lower extremity muscle mass adjusted by height), is confirmed and SMM can be accurately estimated using BIA parameters, a simpler and safer method to prescribe RT without any concerns could be developed. This study aimed to examine the relationship between the BIA measurements and 1RM for leg-press (LP), a typical multi-joint RT exercise of the lower extremities, and to develop BIA-based prediction models of 1RM for LP. Subgroup analysis was also conducted to reveal sex differences in the correlation and accuracy of the BIA-based prediction models of 1RM.

## Results

Forty participants were enrolled in this study. Some participants with a history of injury to the lower limbs (*n* = 1) or lumbar regions (*n* = 1) and those with low back pain (*n* = 3) were excluded. Ultimately, 35 participants (18 men and 17 women) were included in this study. The participants’ characteristics are presented in Table 1. Between the two sexes, men had significantly more muscle mass and higher SMI than women. In addition, the 1RM for LP was significantly higher in men than in women (Table 1).

**Table 1:** Participants’ characteristics.

	All participants (*n* = 35)	Men (*n* = 18)	Women (*n* = 17)	*P*-value
Age, years	28.3 ± 3.8	29.8 ± 3.1	26.7 ± 3.9	0.013
Height, cm	163.3 ± 8.8	170.3 ± 4.5	155.9 ± 5.4	0.0001
Weight, kg	55.5 ± 8.7	61.8 ± 6.5	48.7 ± 4.6	0.0001
BMI, kg/m^2^	20.7 ± 2.1	21.3 ± 2.0	20.1 ± 2.1	0.082
Whole body FM, kg	10.9 ± 3.8	10.0 ± 4.0	11.8 ± 3.6	0.176
Dominant-arm SMM, kg	2.2 ± 0.6	2.8 ± 0.3	1.6 ± 0.2	0.0001
Non-dominant arm SMM, kg	2.2 ± 0.6	2.7 ± 0.3	1.6 ± 0.2	0.0001
Dominant-leg SMM, kg	7.2 ± 1.6	8.5 ± 0.9	5.7 ± 0.6	0.0001
Non-dominant leg SMM, kg	7.2 ± 1.6	8.5 ± 0.9	5.7 ± 0.6	0.0001
Trunk SMM, kg	19.5 ± 3.8	22.8 ± 1.8	15.9 ± 1.4	0.0001
SMI, kg/m^2^	6.9 ± 1.0	7.7 ± 0.5	6.1 ± 0.4	0.0001
1RM for leg-press, kg	71.0 ± 16.5	84.2 ± 8.4	57.1 ± 10.2	0.0001

Data are presented as the mean ± standard deviation.

*1RM* one-repetition maximum, *BMI* body mass index, *FM* fat mass, *SMI* skeletal muscle mass index, *SMM* skeletal muscle mass.

In all participants, the correlation coefficients between 1RM for LP and dominant-leg SMM and 1RM for LP and SMI as a BIA measurement were 0.845 (*P* = 0.0001) and 0.910 (*P* = 0.0001), respectively. In men, the correlation coefficients between 1RM and dominant-leg SMM and 1RM and SMI were 0.527 (*P* = 0.025) and 0.752 (*P* = 0.0001), respectively. Conversely, in women, the correlation coefficients between 1RM and dominant-leg SMM and 1RM and SMI were 0.310 (*P* = 0.225) and 0.613 (*P* = 0.009), respectively (Table 2).

**Table 2:** Correlation analyses between BIA measurements and 1RM for leg-press.

	1RM for leg-press		
	All participants (*n* = 35)	Men (*n* = 18)	Women (*n* = 17)
Dominant-leg SMM	0.845 (*P* = 0.0001)	0.527 (*P* = 0.025)	0.310 (*P* = 0.225)
SMI	0.910 (*P* = 0.0001)	0.752 (*P* = 0.0001)	0.613 (*P* = 0.009)

*1RM* one-repetition maximum, *BIA* bioelectrical impedance analysis, *SMI* skeletal muscle mass index, *SMM* skeletal muscle mass.

**Figure 1:**
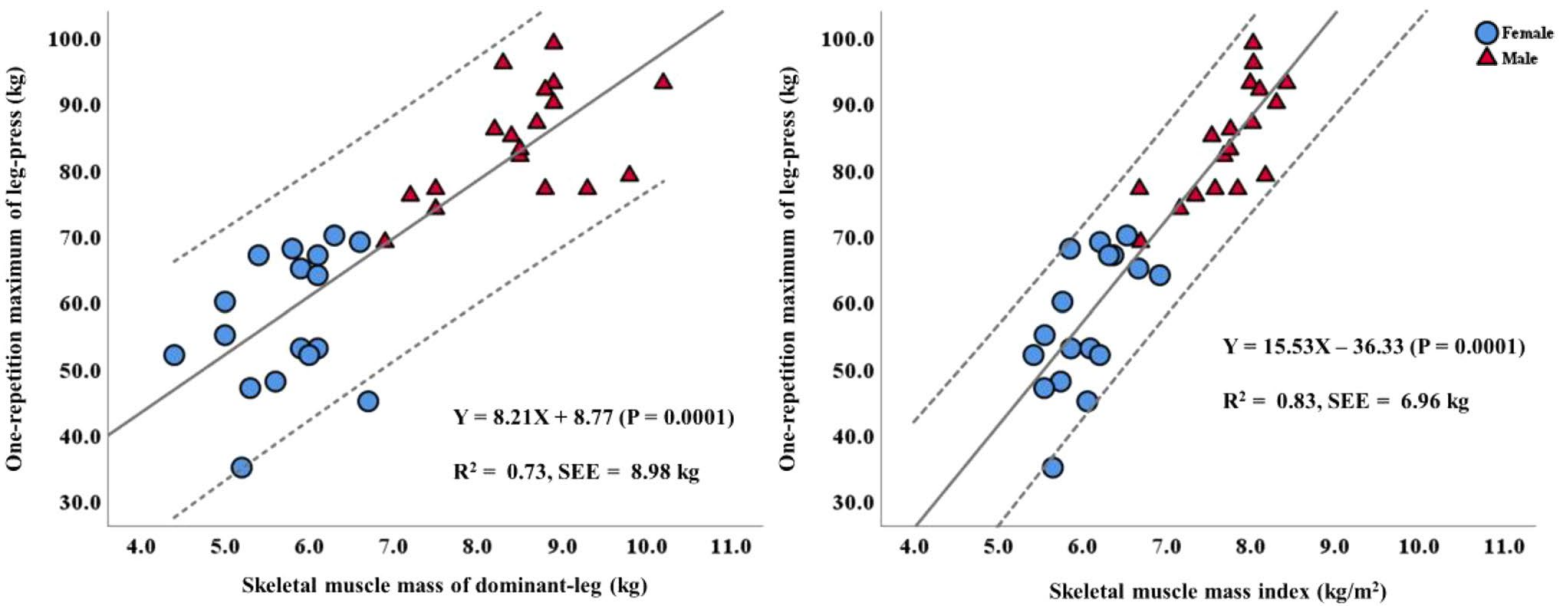
Regression models for one-repetition maximum for leg-press from using BIA measurements. (**a**) Skeletal muscle mass of the dominant leg. (**b**) Skeletal muscle mass index. The dotted lines represent the 95% confidence interval.

The results of the single linear regression analysis are presented in Table 3. Most of the BIA-based prediction models for 1RM for LP were statistically significant except for the model with dominant-leg SMM as an independent variable in women. The R^2^ values of the prediction model using dominant-leg SMM and SMI in all participants were 0.73 (standard error of estimation [SEE]: 8.98 kg, *P* = 0.0001) and 0.83 (SEE: 6.96 kg, *P* = 0.0001), respectively (Fig. 1). In the 1RM prediction model that analyzed sex, R^2^ values in men were 0.28 (SEE: 7.39 kg, *P* = 0.025) in dominant-leg SMM and 0.56 (SEE: 5.73 kg, *P* = 0.0001) in SMI. In women, the R^2^ value of the prediction model with SMI as an independent variable was 0.38 (SEE: 8.29 kg, *P* = 0.0001).

**Table 3:** Prediction models of 1RM for leg-press using BIA measurements.

	Dependent variable	Prediction model	R^2^	SEE	95% confidence interval		*P*-value
					Lower	Upper	
All participants (*n* = 35)	Dominant-leg SMM	Y = 8.21X + 8.77	0.73	8.98	6.798	10.733	0.0001
	SMI	Y = 15.53X − 36.33	0.83	6.96	13.022	18.040	0.0001
Men (*n* = 18)	Dominant-leg SMM	Y = 5.23X + 39.63	0.28	7.39	0.763	9.696	0.025
	SMI	Y = 12.74X − 14.25	0.57	5.73	6.824	18.647	0.0001
Women (*n* = 17)	Dominant-leg SMM	Y = 5.15X + 27.56	0.10	10.0	-3.553	13.831	0.225
	SMI	Y = 14.65X − 31.52	0.38	8.29	4.265	25.039	0.009

*1RM* one-repetition maximum, *BIA* bioelectrical impedance analysis, *SEE* standard error of estimation, *SMI* skeletal muscle mass index, *SMM* skeletal muscle mass.

## Discussion

This study aimed to determine the relationships between the 1RM for LP and BIA measurements and to develop a BIA-based prediction model of 1RM for LP in healthy young adults. We also examined sex differences in the correlational analysis and the prediction models’ accuracy. There were two important findings in this study. First, there were strong correlations between the 1RM for LP and BIA measurements, and accurate prediction may be possible with BIA measurements, particularly with SMI as the dependent variable. Second, there were stronger correlations in men than in women; therefore, accurate 1RM estimation using BIA measurements might be attainable for men.

Although previous studies have revealed that isometric knee extensor muscle strength and BIA measurements are strongly correlated^21,25–27^, few studies analyzed the relationship between 1RM and dynamic muscle strength using BIA measurements. Kanada et al.^28^ reported strong relationships (Rho = 0.70 to 0.78, *P* < 0.01) between the knee-extensor 1RM and each appendicular limb’s SMM or whole-body SMM obtained using BIA in young men. In addition, these authors also reported that 1RM could be accurately estimated by combining isometric muscle strength and SMM in healthy young men^28^. Our results showed stronger correlation coefficients with 1RM for LP and BIA measurements than those in the study of Kanada et al., which might be related to the influence of the required muscle activation in the adopted resistance exercise on BIA measurements. Knee extension exercise mainly requires quadriceps muscle activity^29^, whereas LP requires activities in most lower limb muscles^30^. Therefore, compared with the previous study, our method showed improved results because SMM or SMI, which includes most of the lower limb muscles, might be strongly correlated with and could accurately estimate the 1RM for the multi-joint LP exercise.

In our study, an accurate prediction model was developed when the SMI, an index of upper and lower extremity muscle mass adjusted by height, was used as a dependent variable rather than the dominant-leg SMM. This finding might be explained by the fact that all body parts, including the parts whose strength was directly measured as well as those enabling bodily stabilization at the time of measurement, affect strength assessment. Moreover, a previous study revealed that bodily stabilization by the hands influenced muscle strength production^31^. Since grasping handles is considered a body-stabilization strategy, which is not involved in physiological processes associated with remote muscle contractions^32^, the estimation accuracy of SMM and SMI as dependent variables to predict 1RM for LP might be influenced.

There were differences in the correlation coefficients and accuracy of the estimation model between men and women in this study. In this regard, the correlation between the 1RM for LP and BIA variables or the accuracy of the estimation model was higher in men than in women. A previous study reported a sex difference in the correlation between isometric knee muscle strength and BIA-based SMM in community-dwelling older adults^33^. Our study confirmed the sex differences between 1RM as dynamic muscle strength and BIA measurements and those relationships in healthy young adults. A possible explanation for these results may be the difference in neural determinants of muscle strength between the sexes. While it is well documented that men generally have greater muscle mass than women^34,35^, few studies of sex differences in neural factors are available. However, some studies have revealed that the firing rate of the vastus medialis differed between women and men^36^, and less steady force production in women was caused by unstable modulation of the motor firing discharge rate^37^. Moreover, BIA cannot evaluate such neural factors and only reflects morphological factors, which may explain the differences in the correlation and accuracy of the estimation model between the sexes in this study. Another possible explanation was that BIA could not evaluate the difference in the fiber characteristics of the lower limb muscles between men and women. A previous study revealed that type II fibers, which could produce more force than type I fibers, which are more suitable for continuous force production, are larger in men than in women^38^. Further, BIA could not distinguish muscle fiber types, which might have influenced the correlation and accuracy of the estimation model between the sexes in this study.

Our findings might help athletic trainers or fitness professionals resolve the concerns about the time-consuming and unsafe nature of 1RM measurements. Additionally, these results might potentially be applied in rehabilitation settings, where safety concerns are more important for future studies. Further, our findings might also indicate the necessity for practical equipment that could assess an individual’s body characteristics or capacity more objectively to carry out physical training.

This study had some limitations. First, although sample size calculation was conducted prior to the study initiation, the sample size was too small to draw a clear conclusion regarding the validity and reproductivity of the established prediction models. Hence, a study with a larger sample size should be conducted to confirm our results and their validity and reproductivity in the future. Second, our target population was healthy young adults, not older adults or people with pain or past injuries. Since SMM might not play an important role in muscle strength among healthy older adults^39^ and muscle strength was lower in people with a history of injuries or low back pain compared to those without those problems^40,41^, the correlations and estimation models in our study could not be directly adapted to these populations. To address this issue, multivariable estimation models, adaptive to any population, should be developed in future studies. Finally, since the absolute SMM value reportedly differed among body composition analyzers^42^, the relationship and accuracy of the estimation models may differ from those of other BIA equipment. Therefore, the correlation and accuracy of the estimation model using other equipment should be confirmed in future studies.

In conclusion, 1RM for LP and BIA measurements were strongly correlated, and accurate 1RM prediction from BIA measurements might be attainable in healthy young adults. This methodology might provide a new perspective for sports or fitness experts to resolve the safety and time-consuming concerns for 1RM measurements. The application of our results to rehabilitation medicine might also be expected in future studies.

## Methods

### Study design and ethical approval

This cross-sectional study protocol was approved by the institutional ethics committee of Shinshu University (approval number: 3722). This study was conducted in accordance with the Declaration of Helsinki and was revised in 2013. All participants were informed of the study’s aim, procedures, and potential risks and signed informed consent forms before their participation.

### Study participants

Healthy adults working as medical staff at Kakeyu-Misayama Rehabilitation Center, Kakeyu Hospital, Japan, were conveniently recruited via a displayed poster between July 2017 and November 2017. The inclusion criteria were as follows: (1) age ≥ 20 and < 40 years, (2) no history of injury to the spine or lower limbs, (3) no history of neurological diseases, (4) no pain at rest or during exercise, (5) no pregnancy or possible pregnancy, and (6) no cardiac pacemaker.

### Procedure

First, the body composition was measured using BIA, and then the 1RM was measured. Both assessments were conducted at a fixed time on the same day. Participants were instructed to refrain from eating or drinking large amounts of water 4 h before the measurements and consuming alcohol 8 h before the measurement. Participants were also required not to undertake any intense exercise for 8 h before the measurements.

### BIA measurements

BIA measurements were performed using a portable body composition analyzer Inbody 430 (Biospace, Korea), equipped with a terra-polar eight-point tactile electrode system. It uses three multi-frequencies (5 kHz, 50 kHz, and 250 kHz) to measure the impedance of the participant’s appendicular muscles and trunk for the estimation of the body composition. The measurements by multi-frequencies are considered a better method for assessing muscle function than the single-frequency measurement^26^. A portable body composition analyzer from the Inbody models was confirmed as a reliable and valid tool to assess the SMM in healthy men and women and is considered to have sufficient ability to assess the body composition such as SMM, body fat, and body fat percent like other advanced models^43^. Moreover, the Inbody 430 has been widely used to assess SMM, especially for the Japanese population, in various studies, including large sample cohort studies^44–48^. After the participants wiped their soles off, they stood on the analyzer’s platform, grasping the handles with both hands according to the manufacturer’s guidance. The measurements took approximately 40 s to complete. The analyzer calculated the absolute muscle and fat mass, body fat percentage, and segmental muscle mass values (upper and lower limbs of both sides and trunk). We used dominant-leg SMM and SMI, which was the sum of the appendicular SMM obtained by dividing the participants’ squared height (kg/m^2^), for the analyses because the SMI was reportedly correlated with muscle function in people with sarcopenia^49^.

### 1RM measurement

The 1RM measurement was performed using the participant’s dominant leg with an LP resistance training machine (HUR, Finland). This resistance training machine allowed the participants to lift the loads unilaterally. The 1RM procedure was performed according to the American College of Sports Medicine guidelines^50^. All participants underwent a 5-min warm-up session using an ergo cycle bike before the measurements. The participants sat on the LP machine with their hip and knee joints fixed at approximately 90°, and the pelvis was stabilized by the belt. The participants were also required to hold handgrips placed on both side of the machine seat with each hand. The familiarization session with LP with light resistance for 8–10 repetitions was performed using perceived 50% 1RM. The measurements were started at loads of 80% 1RM. The load in the measurement was progressively changed by 3–10 kg until the participants could not lift the loads. The goal was to complete a maximal lift in five attempts, and 3–5 min of rest were provided between sets. All tests were performed by the same evaluator in the same order.

### Statistical analysis

The sample size analysis was conducted using G* Power software 3.1.9.4 (Heinrich Heine University, Dusseldorf, Germany). Since moderate-to-strong correlations between BIA measurements and isometric muscle strength of the lower limbs have been previously reported^21,25–27^, we set the alpha to 0.05, power to 0.8, and effect size to 0.5 and calculated the required minimum sample size to be *n* = 26. The participants’ characteristics were presented as the mean ± standard deviation. First, we confirmed the normality of the obtained data using the Shapiro–Wilk test, and we also confirmed the homogeneity between the sexes by unpaired t-test. Second, we identified the correlations between each of the variables obtained from the body composition analyzer and the 1RM for LP by calculating Pearson’s product-moment correlation coefficients because the normality of those measurements was confirmed. Finally, to create the 1RM prediction models, a simple linear regression analysis was performed with BIA measurements as independent variables. To evaluate the models’ accuracy, R^2^ and SEE parameters were considered. All analyses were performed using SPSS version 25 (International Business Machine Corp., Armonk, NY, USA). Statistical significance was considered at *P*-values < 0.05.

## Data Availability

The datasets used in this study are available from the corresponding author upon reasonable request.

## References

[1] El-Kotob R (2020). Resistance training and health in adults: an overview of systematic reviews. Appl. Physiol. Nutr. Metab..

[2] Sherrington C (2019). Exercise for preventing falls in older people living in the community. Cochrane Database Syst. Rev..

[3] Haskell WL (2007). Physical activity and public health: updated recommendation for adults from the American College of Sports Medicine and the American Heart Association. Med. Sci. Sports Exerc..

[4] American College of Sports Medicine. American College of Sports Medicine position stand. Progression models in resistance training for healthy adults. *Med. Sci. Sports Exerc.* **41**, 687–708 (2009). 10.1249/MSS.0b013e3181915670 19204579

[5] Borde R, Hortobágyi T, Granacher U (2015). Dose-response relationships of resistance training in healthy old adults: a systematic review and meta-analysis. Sports Med..

[6] Csapo R, Alegre LM (2016). Effects of resistance training with moderate vs heavy loads on muscle mass and strength in the elderly: a meta-analysis. Scand. J. Med. Sci. Sports.

[7] Grgic J, Lazinica B, Schoenfeld BJ, Pedisic Z (2020). Test-retest reliability of the one-repetition maximum (1RM) strength assessment: a systematic review. Sports Med. Open.

[8] Pollock ML (1991). Injuries and adherence to walk/jog and resistance training programs in the elderly. Med. Sci. Sports Exerc..

[9] MacDougall JD, Tuxen D, Sale DG, Moroz JR, Sutton JR (1985). Arterial blood pressure response to heavy resistance exercise. J. Appl. Physiol..

[10] Mayhew JL (1995). Muscular endurance repetitions to predict bench press strength in men of different training levels. J. Sports Med. Phys. Fitness.

[11] Mayhew JL, Johnson BD, Lamonte MJ, Lauber D, Kemmler W (2008). Accuracy of prediction equations for determining one repetition maximum bench press in women before and after resistance training. J. Strength Cond. Res..

[12] Braith RW, Graves JE, Leggett SH, Pollock ML (1993). Effect of training on the relationship between maximal and submaximal strength. Med. Sci. Sports Exerc..

[13] De Witt JK (2018). Isometric midthigh pull reliability and relationship to deadlift one repetition maximum. J. Strength Cond. Res..

[14] Tan AEL, Grisbrook TL, Minaee N, Williams SA (2018). Predicting 1 repetition maximum using handheld dynamometry. PMR.

[15] Niewiadomski W (2008). Determination and prediction of one repetition maximum (1RM): safety considerations. J. Hum. Kinet..

[16] Suchomel TJ, Nimphius S, Bellon CR, Stone MH (2018). The importance of muscular strength: training considerations. Sports Med..

[17] Maughan RJ, Watson JS, Weir J (1983). Strength and cross-sectional area of human skeletal muscle. J. Physiol..

[18] Akagi R (2009). Muscle volume compared to cross-sectional area is more appropriate for evaluating muscle strength in young and elderly individuals. Age Ageing.

[19] O'Brien TD, Reeves ND, Baltzopoulos V, Jones DA, Maganaris CN (2009). Strong relationships exist between muscle volume, joint power and whole-body external mechanical power in adults and children. Exp. Physiol..

[20] Heymsfield SB, Gonzalez MC, Lu J, Jia G, Zheng J (2015). Skeletal muscle mass and quality: evolution of modern measurement concepts in the context of sarcopenia. Proc. Nutr. Soc..

[21] Miyatani M, Kanehisa H, Masuo Y, Ito M, Fukunaga T (2001). Validity of estimating limb muscle volume by bioelectrical impedance. J. Appl. Physiol..

[22] Kim M, Shinkai S, Murayama H, Mori S (2015). Comparison of segmental multifrequency bioelectrical impedance analysis with dual-energy X-ray absorptiometry for the assessment of body composition in a community-dwelling older population. Geriatr. Gerontol. Int..

[23] Chien MY, Huang TY, Wu YT (2008). Prevalence of sarcopenia estimated using a bioelectrical impedance analysis prediction equation in community-dwelling elderly people in Taiwan. J. Am. Geriatr. Soc..

[24] Chen LK (2020). Asian Working Group for Sarcopenia. Asian Working Group for Sarcopenia: 2019 Consensus update on sarcopenia diagnosis and treatment. J. Am. Med. Dir. Assoc..

[25] Yamada Y (2010). Extracellular water may mask actual muscle atrophy during aging. J. Gerontol. A Biol. Sci. Med. Sci..

[26] Yamada Y (2013). Comparison of single- or multifrequency bioelectrical impedance analysis and spectroscopy for assessment of appendicular skeletal muscle mass in the elderly. J. Appl. Physiol..

[27] Yamada Y (2017). The extracellular to intracellular water ratio in upper legs is negatively associated with skeletal muscle strength and gait speed in older people. J. Gerontol. A Biol. Sci. Med. Sci..

[28] Kanada Y (2017). Estimation of 1RM for knee extension based on the maximal isometric muscle strength and body composition. J. Phys. Ther. Sci..

[29] Overend TJ, Cunningham DA, Kramer JF, Lefcoe MS, Paterson DH (1992). Knee extensor and knee flexor strength: cross-sectional area ratios in young and elderly men. J. Gerontol..

[30] Da Silva EM, Brentano MA, Cadore EL, De Almeida APV, Kruel LFM (2008). Analysis of muscle activation during different leg press exercises at submaximum effort levels. J. Strength Cond. Res..

[31] Magnusson SP, Geismar RA, Gleim GW, Nicholas JA (1993). The effect of stabilization on isokinetic knee extension and flexion torque production. J. Athl. Train..

[32] Nuzzo JL, Taylor JL, Gandevia SC (2019). CORP: measurement of upper and lower limb muscle strength and voluntary activation. J. Appl. Physiol..

[33] Hayashida I, Tanimoto Y, Takahashi Y, Kusabiraki T, Tamaki J (2014). Correlation between muscle strength and muscle mass, and their association with walking speed, in community-dwelling elderly Japanese individuals. PLoS ONE.

[34] Janssen I, Heymsfield SB, Wang ZM, Ross R (2000). Skeletal muscle mass and distribution in 468 men and women aged 18–88 yr. J. Appl. Physiol..

[35] Abe T, Kearns CF, Fukunaga T (2003). Sex differences in whole body skeletal muscle mass measured by magnetic resonance imaging and its distribution in young Japanese adults. Br. J. Sports Med..

[36] Peng YL, Tenan MS, Griffin L (2018). Hip position and sex differences in motor unit firing patterns of the vastus medialis and vastus medialis oblique in healthy individuals. J. Appl. Physiol..

[37] Inglis JG, Gabriel DA (2021). Sex differences in the modulation of the motor unit discharge rate leads to reduced force steadiness. Appl. Physiol. Nutr. Metab..

[38] Miller AE, MacDougall JD, Tarnopolsky MA, Sale DG (1993). Gender differences in strength and muscle fiber characteristics. Eur. J. Appl. Physiol. Occup. Physiol..

[39] Beliaeff S, Bouchard DR, Hautier C, Brochu M, Dionne IJ (2008). Association between muscle mass and isometric muscle strength in well-functioning older men and women. J. Aging Phys. Act..

[40] Nadler SF, Malanga GA, De Prince M, Stitik TP, Feinberg JH (2000). The relationship between lower extremity injury, low back pain, and hip muscle strength in male and female collegiate athletes. Clin. J. Sport Med..

[41] Latery PJ, Bruns J, Hiller CE, Nightingale EJ (2017). Relationship between foot pain, muscle strength and size: a systematic review. Physiotherapy.

[42] Yamada M, Yamada Y, Arai H (2016). Comparability of two representative devices for bioelectrical impedance data acquisition. Geriatr. Gerontol. Int..

[43] McLester CN, Nickerson BS, Klisezczewicz BM, McLester JL (2020). Reliability and agreement of various inbody body composition analyzers as compared to dual-energy x-ray absorptiometry in healthy men and women. J Clin Densitom..

[44] Tabara Y (2020). Comparison of diagnostic significance of the initial versus revised diagnostic algorithm for sarcopenia from the Asian Working Group for Sarcopenia. Arch Gerontol Geriatr..

[45] Kitamura E (2021). The relationship between body composition and sleep architecture in athletes. Sleep Med..

[46] Huang CH (2021). A 3-year prospective cohort study of dietary patterns and frailty risk among community-dwelling older adults. Clin. Nutr..

[47] Lyu W, Tanaka T, Son B-K, Akishita M, Iijima K (2022). Associations of multi-faceted factors and their combinations with frailty in Japanese community-dwelling older adults: Kashiwa cohort study. Arch Gerontol Geriatr..

[48] Ishi S (2014). Development of a simple screening test for sarcopenia in older adults. Geriatr Gerontol Int..

[49] Han DS (2016). Skeletal muscle mass adjusted by height correlated better with muscular functions than that adjusted by body weight in defining sarcopenia. Sci. Rep..

[50] Thompson WR, Gordon NF, Pescatello LS (2009). ACSM's Guidelines for Exercise Testing and Prescription.

